# Transcranial direct current stimulation (tDCS) combined with cognitive training in adolescent boys with ADHD: a double-blind, randomised, sham-controlled trial

**DOI:** 10.1017/S0033291721001859

**Published:** 2023-01

**Authors:** Samuel J. Westwood, Marion Criaud, Sheut-Ling Lam, Steve Lukito, Sophie Wallace-Hanlon, Olivia S. Kowalczyk, Afroditi Kostara, Joseph Mathew, Deborah Agbedjro, Bruce E. Wexler, Roi Cohen Kadosh, Philip Asherson, Katya Rubia

**Affiliations:** 1 Department of Child & Adolescent Psychiatry, King's College London, London, UK; 2 School of Psychology, University of Surrey, Guildford, UK; 3 Department of Neuroimaging, King's College London, London, UK; 4 Department of Biostatistics, King's College London, London, UK; 5 Department of Psychiatry, Yale School of Medicine, New Haven, CT, USA; 6 Department of Experimental Psychology, University of Oxford, Oxford, UK; 7 Social Genetic & Developmental Psychiatry, King's College London, London, UK

**Keywords:** ADHD, randomised controlled trial, tDCS, treatment

## Abstract

**Background:**

Transcranial direct current stimulation (tDCS) could be a side-effect-free alternative to psychostimulants in attention-deficit/hyperactivity disorder (ADHD). Although there is limited evidence for clinical and cognitive effects, most studies were small, single-session and stimulated left dorsolateral prefrontal cortex (dlPFC). No sham-controlled study has stimulated the right inferior frontal cortex (rIFC), which is the most consistently under-functioning region in ADHD, with multiple anodal-tDCS sessions combined with cognitive training (CT) to enhance effects. Thus, we investigated the clinical and cognitive effects of multi-session anodal-tDCS over rIFC combined with CT in double-blind, randomised, sham-controlled trial (RCT, ISRCTN48265228).

**Methods:**

Fifty boys with ADHD (10–18 years) received 15 weekday sessions of anodal- or sham-tDCS over rIFC combined with CT (20 min, 1 mA). ANCOVA, adjusting for baseline measures, age and medication status, tested group differences in clinical and ADHD-relevant executive functions at posttreatment and after 6 months.

**Results:**

ADHD-Rating Scale, Conners ADHD Index and adverse effects were significantly lower at post-treatment after sham relative to anodal tDCS. No other effects were significant.

**Conclusions:**

This rigorous and largest RCT of tDCS in adolescent boys with ADHD found no evidence of improved ADHD symptoms or cognitive performance following multi-session anodal tDCS over rIFC combined with CT. These findings extend limited meta-analytic evidence of cognitive and clinical effects in ADHD after 1–5 tDCS sessions over mainly left dlPFC. Given that tDCS is commercially and clinically available, the findings are important as they suggest that rIFC stimulation may not be indicated as a neurotherapy for cognitive or clinical remediation for ADHD.

## Introduction

Attention-deficit/hyperactivity disorder (ADHD) is characterised by persisting, age-inappropriate and impairing symptoms of inattention and/or impulsivity-hyperactivity (American Psychiatric Association, 2013). ADHD is also associated with deficits in executive functions (EF), most prominently in motor and interference inhibition, sustained attention and vigilance, switching, working memory and timing (Coghill, Toplak, Rhodes, & Adamo, 2018; Rubia, 2018). Neuroimaging studies indicate delayed structural and functional brain maturation (Shaw et al., 2007; Sripada, Kessler, & Angstadt, 2014), and consistent underactivation in predominantly right inferior frontal (rIFC), dorsolateral prefrontal (dlPFC) and anterior cingulate cortices, as well as striatal, parietal and cerebellar regions during cognitive control, timing and attention tasks (Hart, Radua, Mataix-Cols, & Rubia, 2012, 2013; Lukito et al., 2020; Norman et al., 2016; Rubia, 2018).

Psychostimulants are the gold-standard treatment for improving ADHD symptoms, but have possible associated side effects (Cortese, 2020), poor adherence in adolescence (Cortese et al., 2018; Cunill, Castells, Tobias, & Capellà, 2016) and are not indicated for all individuals with ADHD (Faraone et al., 2015). Evidence of longer-term efficacy is also limited (Cortese et al., 2018; Swanson et al., 2018), possibly due to brain adaptation (Fusar-Poli, Rubia, Rossi, Sartori, & Balottin, 2012). Meta-analyses indicate small to moderate efficacy of behavioural therapies, cognitive training (CT), neurofeedback or dietary interventions on ADHD symptoms (Catalá-López et al., 2017). Non-invasive brain stimulation techniques, however, are promising given their potential to stimulate key dysfunctional brain regions associated with ADHD, with potentially longer-term neuroplastic effects that drugs cannot offer (Cinel, Valeriani, & Poli, 2019; Rubia, 2018; Sierawska et al., 2019; STIPED Press Release, 2017; Westwood, Radua, & Rubia, 2020). Transcranial direct current stimulation (tDCS) is particularly well-suited for paediatric populations as it is user-friendly, well tolerated with minimal side effects (Bikson et al., 2016), and is cheaper relative to other techniques, such as transcranial magnetic stimulation (Gilbert, 2019).

In tDCS, a weak direct electrical current is delivered through two electrodes placed on the scalp (one anode, one cathode), generating subthreshold, polarity-dependent shifts in resting membrane potentials in underlying brain regions. The resulting net increase (predominantly under the anode) or decrease (predominantly under the cathode) in neuronal excitability leads to modulation of the neuronal network (Liu et al., 2018), with neurophysiological effects persisting after stimulation, presumably by potentiating mediators of practice-dependent synaptic plasticity, including GABA, glutamate (Filmer, Ehrhardt, Bollmann, Mattingley, & Dux, 2019; Stagg & Nitsche, 2011), dopamine and noradrenaline (Fonteneau et al., 2018; Kuo, Paulus, & Nitsche, 2008; Monte-Silva, Liebetanz, Grundey, Paulus, & Nitsche, 2010).

Evidence of cognitive performance and clinical improvement following tDCS is, however, limited. Two meta-analyses of tDCS studies stimulating mainly the left dlPFC in 1–5 sessions in children and adolescents with ADHD indicate a modest improvement in inhibitory control measures (Salehinejad, Wischnewski, Nejati, Vicario, & Nitsche, 2019) with the latter one showing non-significant improvement on processing speed and inhibitory measures with no effects on attention measures (Westwood et al., 2020). Only two sham-controlled studies tested ADHD symptoms, reporting improvement in inattentive symptoms, but not impulsivity/hyperactivity, immediately, 1 (Cachoeira et al., 2017; Soff, Sotnikova, Christiansen, Becker, & Siniatchkin, 2017) and 2 weeks (Cachoeira et al., 2017) after anodal tDCS over left or right dlPFC.

There is evidence that tDCS effects can be enhanced when combined with CT by functionally priming the brain regions that mediate the cognitive function being trained (Au et al., 2016, 2017; Katz et al., 2017; Kronberg, Bridi, Abel, Bikson, & Parra, 2017). Multi-session anodal tDCS combined with CT has also been shown to elicit longer-term cognitive improvements in healthy controls for up to 1 (Jones, Stephens, Alam, Bikson, & Berryhill, 2015; Stephens & Berryhill, 2016), nine (Ruf, Fallgatter, & Plewnia, 2017) or 12 months (Katz et al., 2017), and clinical improvements in psychiatric disorders for at least 1 month (Kekic, Boysen, Campbell, & Schmidt, 2016; Moffa, Brunoni, Nikolin, & Loo, 2018; Tortella et al., 2015), suggesting longer-term neuroplastic effects.

Most studies in ADHD have targeted the left dlPFC. However, one of the most consistent findings of meta-analyses of functional magnetic resonance imaging (fMRI) studies is the underactivation of rIFC during tasks of cognitive control (Hart, Radua, Nakao, Mataix-Cols, & Rubia, 2013; Lukito et al., 2020; McCarthy, Skokauskas, & Frodl, 2014; Norman et al., 2016; Rubia, 2018). The rIFC is a cognitive control ‘hub’ region, playing a key role in cognitive control (Aron, Robbins, & Poldrack, 2014; Langner, Leiberg, Hoffstaedter, & Eickhoff, 2018), sustained attention (Kim, 2014; Langner & Eickhoff, 2013; Petersen & Posner, 2012) and timing networks (Radua, del Pozo, Gómez, Guillen-Grima, & Ortuño, 2014), mediating key functions of impairment in ADHD (Coghill et al., 2018; Noreika, Falter, & Rubia, 2013). The rIFC dysfunction has also been shown to be disorder-specific to ADHD compared to several neurodevelopmental disorders in comparative fMRI meta-analyses of cognitive control (Lukito et al., 2020; Norman et al., 2016; Rubia, 2018). Further, its upregulation is the most consistent effect of single-dose and longer-term psychostimulant medication (Norman et al., 2016; Rubia et al., 2014).

Only three published studies applied tDCS over rIFC in ADHD, in one or five sessions. One found improvements after anodal relative to sham tDCS in Flanker task errors and intrasubject response variability in 21 adolescents with ADHD (Breitling et al., 2016), but no effect in 14 children with ADHD on a combined n-back and Stop task with high definition or conventional anodal tDCS (Breitling et al., 2020). In 20 undiagnosed high-school students with ADHD symptoms, anodal compared to sham tDCS improved Go accuracy in a Go/No-Go (GNG) task but no other inhibitory or Stroop task measures (Soltaninejad, Nejati, & Ekhtiari, 2015b). To our knowledge, no sham-controlled study so far has measured clinical and cognitive effects of multi-session anodal tDCS over rIFC in combination with CT in patients with ADHD

In this double-blind, sham-controlled, randomised controlled trial (RCT), 50 children and adolescents with ADHD were administered 15 consecutive weekday sessions of anodal or sham tDCS over rIFC combined with CT of EF typically impaired in ADHD. The primary outcome measures were parent-rated clinical symptoms and cognitive performance in an inhibition and a sustained-attention task. Secondary outcome measures were other clinical, safety and EF measures.

Given some evidence of clinical and cognitive improvements with anodal tDCS over the dlPFC (Cachoeira et al., 2017; Soff et al., 2017) or rIFC (Breitling et al., 2016; Soltaninejad, Nejati, & Ekhtiari, 2015a) and prolonged effects when tDCS is paired with CT, we hypothesised that, compared to sham, at posttreatment, multi-session anodal tDCS of rIFC with multi-EF training would show greater improvement in ADHD symptoms and/or performance on EF tasks mediated by rIFC and targeted by the CT. We also hypothesised that clinical and EF task improvement would persist 6 months after posttreatment. Finally, we hypothesised no side or adverse effects.

## Materials and methods

### Trial design

This pre-registered (ISRCTN48265228), double-blind, sham-controlled, parallel RCT compared multi-session anodal *v.* sham tDCS over the rIFC combined with multi-EF training. Randomisation to stimulation group was stratified by age (10–14.5 and 14.5–18 years) and medication status (naïve and non-naïve) using Smith randomisation (Kaiser, 2012; Smith, 1984) conducted by Innosphere Ltd (Haifa, Israel). Experimenters, participants and parents/caregivers were blind to stimulation group. This trial received local research ethics committee approval (RECID: 17/LO/0983) and was conducted in accordance with the Declaration of Helsinki and Consolidated Standards of Reporting Trials (CONSORT) guidelines (Moher et al., 2010).

### Participants

**Fig. 1.**
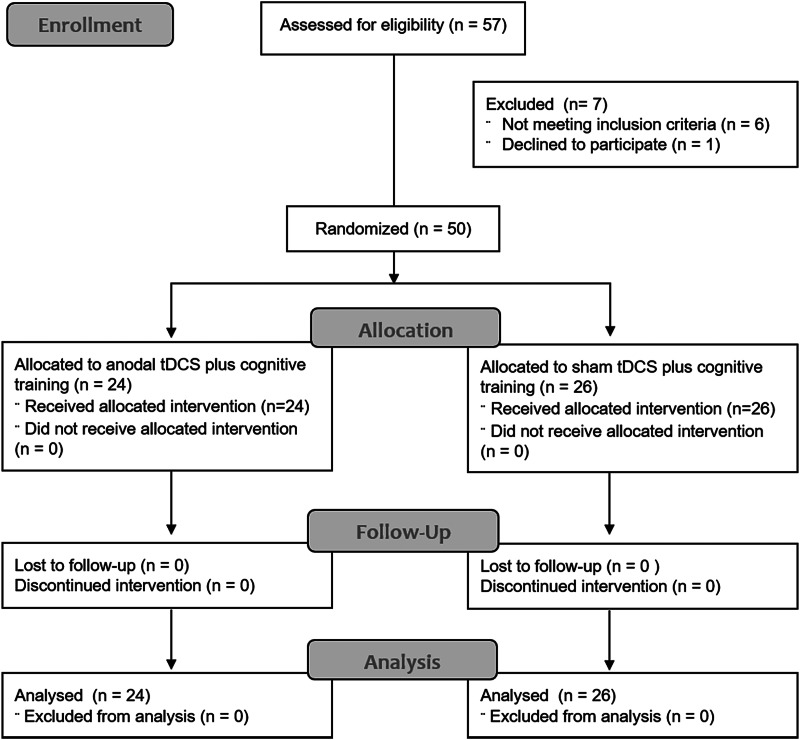
CONSORT flow diagram (Moher et al., 2010) of this RCT from enrolment, intervention allocation, follow-up and analysis.

Fifty male participants (10–18 years) were recruited from South London clinics, social media and parent support groups (February 2018–20). All participants had a clinical DSM-5 diagnosis of ADHD established by a clinician, and validated by the Schedule of Affective Disorders and Schizophrenia for School-Age Children-Present and Lifetime version (K-SADS-PL; DSM-IV) (Kaufman et al., 1997) and the Conners 3rd Edition–Parent (Conners 3-P, cut-off *t* score > 60) (Conners, Pitkanen, & Rzepa, 2011). Autism spectrum disorder (ASD) was excluded using a combination (both required) of both the parent-rated Social Communication Questionnaire (SCQ, cut-off > 17) (Eaves, Wingert, Ho, & Mickelson, 2006) and the pro-social scale of the Strengths and Difficulties Questionnaire (SDQ, cut-off < 5) (Goodman, 1997); for participants falling outside these criteria, the absence of ASD was confirmed by their clinician. Further exclusion criteria were IQ below 80 (Wechsler Abbreviated Scale of Intelligence, WASI-II) (Wechsler, 2011), history of alcohol or substance abuse, neurological illness, comorbid major psychiatric disorders as established by the K-SADS-PL or clinical diagnoses [except for conduct disorder/oppositional defiant disorder (ODD)], contraindications to tDCS [e.g. metallic implants (except dental braces), previous neurosurgical procedures, history of migraine, diseased/damaged skin on the scalp], treated with drugs that lower seizure thresholds (e.g. antipsychotics, tricyclic antidepressants), and an inability to provide consent from the legal caregiver for children under 16 years or from participants over 16 years (Fig. 1). Participants received £540 plus travel expenses for participating.

Baseline assessment was scheduled at least 2 weeks after medication titration. Thirty-two participants received stable ADHD medication (non-psychostimulants: 2; psychostimulants: 30; between 21 weeks and 10 years). To help avoid medication effects from masking tDCS effects, 14 of the 32 participants followed our optional request to abstain from psychostimulants at least 24 h before each assessment time point (range: 24–72 h), of which eight abstained only 24 h beforehand (sham, 4; anodal tDCS, 4). A further seven participants abstained for trial duration (i.e. baseline to posttreatment). One participant stopped taking medication 3 months before follow-up.

### Intervention

**Fig. 2.**
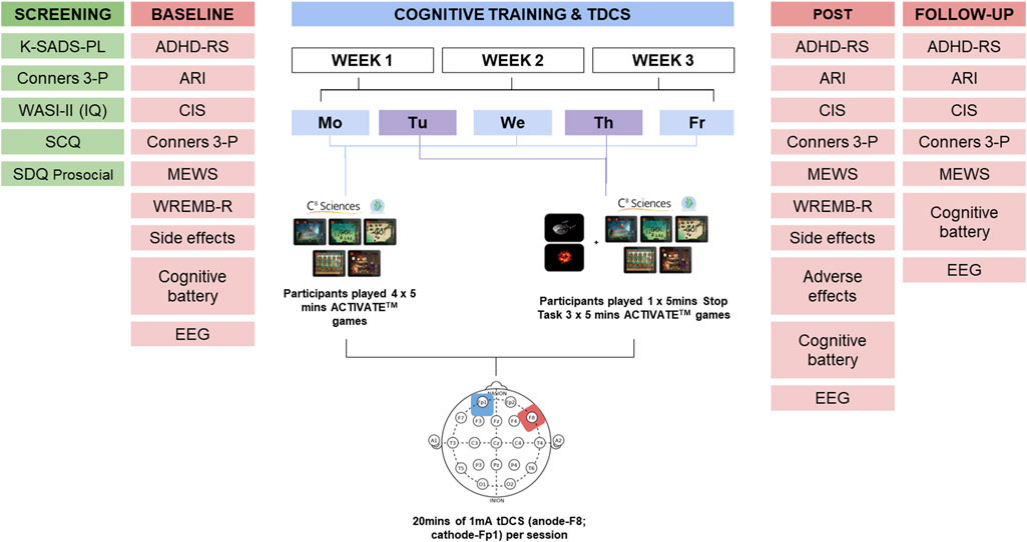
Schematic overview of the study design. ADHD-RS, Attention Deficit Hyperactivity Disorder-Rating Scale; ARI, Affective Reactivity Index; CIS, Columbia Impairment Scale; Conners 3-P, Conners' 3rd Edition Parent Rating; Cognitive battery, Maudsley Attention and Response Suppression task battery, vigilance, Wisconsin Card Sorting Task, visual-spatial working memory, verbal fluency; K-SADS-PL, Kiddie-SADS-Present and Lifetime Version; MEWS, Mind Excessively Wandering Scale; SCQ, Social Communication Questionnaire (Lifetime), SDQ, Social Difficulties Questionnaire (prosocial scale only); WASI-II, Wechsler Abbreviated Scale of Intelligence, 2nd Edition; WREMB-R, Weekly Rating of Evening and Morning Behavior-Revised.

Over 15 consecutive weekdays, participants played four 5 min CT games during 20 min of anodal or sham tDCS. CT was composed of four ACTIVATE^TM^ (Wexler et al., 2016) games (Monday, Wednesday and Friday) or a Stop task (Rubia, Smith, & Taylor, 2007) followed by three ACTIVATE^TM^ games (Tuesday and Thursday). ACTIVATE^TM^ games were played on an iPad Air 2 10.2″, the Stop task on a Dell XPS 15.6″ Touch Laptop (Fig. 2).

#### CT games

ACTIVATE^TM^ trains multiple EF with engaging video games that increase in complexity as participants improve. Five of the six games were selected to target ADHD-relevant EF, including selective and multiple simultaneous attention, inhibition and cognitive flexibility (i.e. *Magic Lens*, *Peter's Printer Panic*, *Treasure Trunk*) and visual-spatial working memory (i.e. *Grub Ahoy*, *Monkey Trouble*). Before each game, participants chose a game to play from three options; the ACTIVATE^TM^ programme tracked choices and constrained future options so that multiple EF were twice as likely to be trained as working memory (Wexler et al., 2016).

The training tracking Stop task trained motor response inhibition by requiring participants to cancel a prepotent motor response to a go signal that is followed shortly by an unexpected and rare (30% of trials) stop signal (Rubia et al., 2007). The delay between go and stop signals is dynamically adjusted to the participant performance, with better inhibition resulting in longer stop signal delays thus increasing the difficulty to withhold a response. Participants were encouraged to wait for the stop signal before responding to the go signal, thereby training inhibition and waiting behaviour/response delay (see online Supplementary material).

#### tDCS

Direct current was delivered by NovoStim (InnoSphere, Haifa, Israel) via a pair of 25 cm^2^ brush electrodes^†^^1^ dipped in saline solution (0.45 mol). Using the EEG 10–20, the anode was placed over rIFC (F8) (Okamoto et al., 2004; Okamoto & Dan, 2005), the cathode over the contralateral supraorbital area (Fp1), and held in place by a 10–20 EEG cap. For anodal tDCS, 1 mA was administered for 20 min (current density: 0.04 cm^2^; total charge: 0.8 C/cm^2^) with a 30 s fade-in/fade-out. For sham stimulation, only the 30 s fade-in/fade-out was administered (Palm et al., 2013). After each week, participants, parents and tDCS administrators were asked to guess if participants had received anodal or sham tDCS.

### Primary outcomes

#### Cognitive

The adult version of the Maudsley Attention and Response Suppression (MARS) task battery (Penadés et al., 2007; Rubia et al., 2007) measured motor response inhibition [GNG; dependent variable (DV): probability of inhibition (PI)] and sustained attention [Continuous Performance Task (CPT); DV: omission and commission errors] (see online Supplementary material).

#### ADHD symptoms

Parent-rated ADHD Rating Scale-IV (ADHD-RS) Home Version (DV: Total Scores) (DuPaul, Power, Anastopoulos, & Reid, 1998), a standard measure of treatment effects in DSM-IV ADHD symptoms.

### Secondary outcomes

#### Cognitive measures

MARS tasks (Penadés et al., 2007; Rubia et al., 2007) measured interference inhibition (Simon Task; DV: Simon reaction time effect) or time discrimination (Time Discrimination Task; DV: percentage correct). The Mackworth Clock Task (Lichstein, Riedel, & Richman, 2000; Mackworth, 1948; PsyTookit, 2017) measured vigilance (DV: percentage omissions and commission errors), the Wisconsin Card Sorting Task (PsyToolkit, 2018) (WCST; DV: total and preservative errors) measured cognitive flexibility, and the C8 Sciences version of the NIH List Sorting Working Memory (WM) Task (DV: total score) (Tulsky et al., 2014) measured visuo-spatial working memory. Phonetic and semantic fluency (DV: percentage of correct responses) (Troyer, 2000) were measured to account for potential downregulation of lIFC mediated language production functions via interhemispheric inhibition.

#### Generic EF

Three intercorrelated generic, task-independent EF were measured, which included: (1) general speed of information processing [i.e. mean reaction times (MRT) for Go (GNG), Congruent (Simon) and Target (CPT) trials, weighted by number of trials] (2); intrasubject response variability [i.e. Coefficient of Variance (CV; s.d. of MRT divided by MRT) for Go (GNG), Congruent (Simon) and Target (CPT) trials]; and (3) prematurity (i.e. premature responses to all trials in GNG, Simon and CPT) (online Supplementary Tables S1–S5 and Figs S1–S3).

#### ADHD symptoms and related impairments

Caregiver-rated Conners 3-P ADHD Index (Conners et al., 2011) measured ADHD symptoms, Weekly Parent Ratings of Evening and Morning Behaviour-Revised scale (WREMB-R) (Wehmeier, Dittmann, Schacht, Helsberg, & Lehmkuhl, 2009) and the Columbia Impairment Scale-Parent version (CIS) (Bird et al., 1993) measured ADHD-related difficulties and functional impairments, the child- and caregiver-rated Affective Reactivity Index (ARI) measured irritability (Stringaris et al., 2012), and the child-rated Mind Excessively Wandering Scale (MEWS) measured mind-wandering (Mowlem et al., 2019).

#### Safety, feasibility and tolerability measures

Safety was measured with parent-rated questionnaires on side effects (Hill & Taylor, 2001) and adverse events (Döpfner, Lehmkuhl, & Steinhausen, 2006). Participants rated their mood and wakefulness via a visual analogue scale (Alegria et al., 2017; Maurizio et al., 2014) and tDCS tolerability (Antal et al., 2017). Parents and participants rated the overall impression of tDCS and CT using a rating scale from 0 (never) to 4 (always) designed for this study.

#### EEG

EEG was measured at rest and during the GNG task at each assessment timepoint but will be reported in a separate publication.

#### Training outcome measures

The ACTIVATE^TM^ DV was the highest game level reached each week averaged across three games that loaded on multiple EF (i.e. *Treasure Trunk*, *Magic Lens*, *Printer Panic*). The Stop task DV was PI on average for each week.

### Assessment time points

Primary and secondary outcome measures were collected at baseline, posttreatment and 6-month follow-up. Adverse events and overall impression of tDCS and CT were measured at posttreatment. Ratings of mood, wakefulness and the tolerability of tDCS were measured in each stimulation session. Baseline and posttreatment sessions were scheduled up to 3 weeks before the first and after the last stimulation session, respectively.

### Statistical methods

#### Confirmatory analysis

Repeated-measures analysis of covariance (ANCOVA) tested group differences across posttreatment and follow-up, covarying for age in months, medication status (naïve, on- or off-medication) and baseline value of outcomes where applicable. Sensitivity analyses removed statistical outliers on cognitive tasks, participants who changed medication between posttreatment and follow-up, or participants whose treatment spanned over 4 not 3 weeks (online Supplementary Tables S6–S8). The *α* level was 0.05, and corrected for multiple testing with False Discovery Rate (FDR) with Benjamini–Hochberg adjustment (Benjamini & Hochberg, 1995). Only results with adjusted *p* values are reported (for unadjusted, see Tables 2–3). Analyses were conducted with IBM SPSS Statistics 26 (IBM Corp., Armonk, NY, USA).

#### Medication status

Medication status was coded as a categorical covariate with three groups: medication-naïve, on-medication and off-medication. Participants who abstained for the treatment trial period were coded as off-medication, participants who abstained at assessment time points only were coded as off-medication for cognitive outcomes and on-medication for clinical outcomes (which can cover 3 weeks).

#### Missing data and statistical outliers

In primary and secondary outcomes, only posttreatment WM task data for one participant were missing. Missing stop signal task data (1.33%) were random and replaced by group averages.

#### Power analysis

An *a priori* power analysis conducted prior to trial registration indicated that a minimum of 40 participants would be adequate to detect a meaningful effect on clinical and cognitive measures (Ditye, Jacobson, Walsh, & Lavidor, 2012; Jacobson, Ezra, Berger, & Lavidor, 2012).

## Results

### Baseline demographics

Compared to sham tDCS, the anodal tDCS group was significantly younger; had fewer years in education; higher ADHD-RS Total Scores and ODD symptoms; worse performance on the Macworth Clock, Time Discrimination and list sort working memory tasks; and spent more time playing *Peter's Printer Panic* (Table 1).

**Table 1.** Baseline demographic, clinical, cognitive measures and medication status; the number of tDCS and CT sessions; and the time spent playing each CT game in the sham and anodal tDCS groups

	Mean (s.d.)		Independent samples *t* test	
	Sham tDCS	Anodal tDCS	*t*(1, 48)	*p*
Demographics				
*N*	26	24		
Mean age in years	**14.23 (2.06)**	**13.05 (1.98)**	**−2.06**	**0.04**
IQ (WASI-II)	105.15 (13.83)	100.08 (13.17)	−1.33	0.19
Years of education	**9.81 (2.08)**	**8.63 (2)**	**−2.05**	**0.05**
Age of onset of ADHD (years)	4.85 (3.21)	4.42 (2.92)	−0.49	0.62
SCQ	8.31 (7.71)	10.04 (6.23)	0.87	0.39
SDQ (prosocial)	7.31 (2.15)	6.5 (2.67)	−1.18	0.24
Kiddie-SADS-Present and Lifetime Version (ADHD module)				
Total number of ADHD symptoms	12.04 (2.49)	12.83 (3.24)	0.98	0.33
Inattention symptoms	7.58 (0.86)	7.58 (1.10)	0.02	0.98
Hyperactivity/impulsivity symptoms	4.39 (2.40)	5.25 (2.52)	1.24	0.22
Oppositional defiant disorder symptoms	**7 (27%)**	**13 (54%)**	**2.40**	**0.02**
*Clinical measures*				
ADHD-RS				
ADHD-RS Total Score	**37.08 (7.14)**	**41.71 (8.13)**	**2.14**	**0.04**
ADHD-RS Inattention	21.39 (4.61)	23.21 (3.68)	1.54	0.13
ADHD-RS Hyperactivity/Impulsivity	15.69 (4.77)	18.50 (5.97)	1.84	0.07
Conners 3-P (T-Score)				
ADHD Index	14.35 (3.89)	16.25 (3.99)	1.71	0.09
Global Index	83.42 (7.75)	84.71 (7.43)	0.60	0.55
DSM-5 Inattention	82.46 (8.32)	85.00 (7.16)	1.52	0.26
DSM-5 Hyperactivity/Impulsivity	83.50 (9.84)	85.88 (7.95)	0.93	0.36
ARI – raw scores				
Parent-rated	0.83 (0.51)	0.92 (0.58)	0.63	0.53
Child-rated	0.64 (0.48)	0.81 (0.51)	1.26	0.21
MEWS	16.27 (8.30)	18.71 (7.52)	1.09	0.28
WREMB-R Total Score	20.81 (5.53)	23.58 (5.59)	1.76	0.08
CIS	21.81 (7.85)	24.67 (9.31)	1.18	0.25
Side effects	13.69 (5.90)	18.96 (12.19)	1.96	0.06
*Cognitive measures*				
Primary outcomes				
Go/No-Go Task	49.80 (19.46)	46.20 (23.72)	−0.59	0.59
Probability of inhibition (*%*)				
Continuous Performance Task				
Omissions (%)	13.59 (14.70)	15.69 (13.75)	0.52	0.60
Commissions (%)	1.90 (3.2)	2.67 (2.78)	0.92	0.37
Secondary outcomes				
Simon Task	63.39 (30.38)	80.17 (45.30)	1.55	0.13
Simon RT effect				
Time Discrimination Task				
Total correct (%)	76.80 (15.78)	68.54 (12.85)	−2.02	**0.05**
Macworth Clock Task				
Omissions (%)	34.42 (18.47)	49.27 (18.03)	2.87	**0.006**
Commissions (%)	3.94 (6.4)	8.61 (9.04)	2.12	**0.04**
Wisconsin Card Sorting Task				
Perseverative errors	14.89 (4.60)	14.08 (4.84)	−0.60	0.551
Non-preservative errors	9.31 (4.2)	7.67 (3.74)	−0.57	0.57
List Sort Working Memory Task				
Total score	31.08 (11.60)	19.92 (13.45)	−3.15	**0.003**
Verbal Fluency Task				
Letter (% correct)	93.42 (7.26)	91.31 (8.47)	−0.95	0.35
Semantic (% correct)	94.91 (6.98)	95.25 (4.99)	0.19	0.85
Speed of Processing				
Mean reaction times	368.64 (34.95)	381.79 (48.12)	1.11	0.27
Response Variability				
Intrasubject coefficient of variation	0.27 (07)	0.31 (0.07)	1.95	0.06
Prematurity				
Premature responses	24.244 (46.64)	24.36 (25.37)	0.01	0.99
Cognitive training				
No. of completed tDCS and CT sessions^a^	14.85 (0.78)	15.00 (0.00)	0.96	0.34
Total game play (mins)	265.96 (15.55)	269.17 (5.84)	1.18	0.24
Grub Ahoy	24.62 (10.76)	24.79 (11.74)	0.06	0.96
Magic Lens	58.46 (13.17)	55.63 (15.90)	0.69	0.49
Monkey Trouble	26.04 (11.22)	27.5 (14.78)	0.39	0.70
Peter's Printer Panic	92.69 (101.88)	101.88 (12.14)	2.29	**0.03**
Treasure Trunk	62.69 (19.25)	60.83 (18.92)	0.34	0.73
			Pearson χ^2^	
	*N* (%)			
			χ^2^ (3)	*p*
ADHD medication status				
Medication-naïve	8 (31%)	10 (42%)	6.28	0.10
On medication	3 (27%)	8 (33%)		
On-medication except for assessments	10 (39%)	4 (17%)		
Off medication	5 (19%)	2 (8%)		
			χ^2^ (5)	*p*
ADHD medication type				
Atomoxetine	0	1 (4%)	7.54	0.18
Dexamfetamine	0	1 (4%)		
Guanfacine	0	2 (8%)		
Lisdexamfetamine	2 (8%)	0		
Methylphenidate	16 (62%)	10 (42%)		
Medication-naïve	8 (31%)	10 (42%)		

ADHD-RS, Caregiver-rated ADHD Rating Scale; ARI, Affective Reactivity Index; CIS, Columbia Impairment Scale-Parent; MEWS, Mind Excessively Wandering Scale; s.d., standard deviation; SCQ, Social Communication Questionnaire; SDQ, Social Difficulties Questionnaire; WASI-II, Wechsler Abbreviated Scale of Intelligence; WREMB-R, Weekly Parent Ratings of Evening and Morning Behaviour-Revised. Significance group differences are highlighted in bold.

a One participant could not attend three stimulation sessions due to extreme weather conditions.

### Primary outcome measures

#### Cognitive

There were no significant effects on primary cognitive outcome measures after adjusting for multiple testing (Table 2; online Supplementary Fig. S4).

**Table 2.** Summary of adjusted average performance on primary cognitive and clinical outcome measures after sham and anodal tDCS combined with CT

	Posttreatment		Follow-up		ANCOVA					
Primary outcomes	Sham tDCS	Anodal tDCS	Sham tDCS	Anodal tDCS	Time		Group		Time by group	
	Adjusted mean (s.d.)^a^				*F*_(1,44)_	*p*^b^	*F*_(1,44)_	*p*^b^	*F*^(*1,44*)^	*p*^b^
Cognitive	*N* *=* 26	*N* *=* 24	*N* *=* 26	*N* *=* 24						
Go/No-Go Task										
PI (%)	54.18 (14.18)	55.48 (14.23)	56.95 (16.96)	58.41 (17.02)	0.01	0.93 (0.93)	0.12	0.73 (0.73)	0.001	0.97 (0.97)
Continuous Performance Task										
Omission (%)	9.88 (8.44)	14.36 (8.48)	8.31 (8.22)	11.70 (8.25)	0.32	0.58 (1.00)	3.87	**0.06 (0**.09**)**	0.15	0.71 (1.00)
Commission (%)	1.04 (1.65)	2.16 (1.66)	0.97 (1.14)	1.26 (1.14)	0.08	0.79 (1.00)	4.96	0**.03 (0**.09**)**	2.67	0.14 (0.42)
*Clinical*										
ADHD-RS^c^										
Total score	25.55 (8.91)	32.48 (8.96)	31.58 (9.51)	27.91 (9.54)	1.28	0.26	0.57	0.46	10.58	**0.002**
Inattention	14.61 (5.04)	18.01 (5.05)	16.87 (5.35)	16.10 (5.34)	0.04	0.84	1.41	0.24	4.02	**0.051**
Hyperactivity/impulsivity	11.30 (4.95)	14.10 (4.95)	14.62 (5.00)	11.91 (5.00)	0.01	0.91	0.001	0.97	13.08	**0.001**

ADHD-RS, ADHD Rating Scale; PI, probability of inhibition; s.d., standard deviation.

Benjamini–Hochberg adjusted *p* values given in parentheses.

a Adjusted values as predicted by the repeated-measures ANCOVA testing group differences at posttreatment and follow-up, adjusting for baseline, age at entry and medication status (naïve, off-medication, on-medication).

b Benjamini–Hochberg adjustment was applied to *p* values for time, group and time by group interaction effect separately and was applied separately to primary cognitive, secondary cognitive and secondary clinical outcome measures separately.

c Benjamini–Hochberg adjustment was not applied to these measures.

#### ADHD symptoms

There was only a significant time by group interaction (*F*_1,44_ = 10.58, *p* = 0.002, *η*p^2^ = 0.19); simple effects analysis showed that the anodal *v.* sham tDCS group had higher scores at posttreatment but not at follow-up [posttreatment: *p* = 0.011 (95% CI 1.65–12.21); follow-up: *p* = 0.20 (95% CI −9.30 to 1.97)]. To determine what drove this effect, exploratory simple effects analysis of subscales showed that the anodal *v.* sham tDCS group had higher scores at posttreatment on Inattention (*p* = 0.03) and Hyperactivity-Impulsivity subscales (*p* = 0.06), with the latter being lower for active *v.* sham tDCS at follow-up (*p* = 0.07) (Table 2; online Supplementary Fig. S5).

### Secondary outcomes measures

#### Cognitive

The were no significant effects on secondary cognitive outcome measures after adjusting for multiple testing (Table 3).

**Table 3.** Summary of adjusted average performance on secondary cognitive and clinical outcome measures after sham and anodal tDCS combined with CT

	Posttreatment		Follow-up		ANCOVA					
Secondary outcomes	Sham tDCS	Anodal tDCS	Sham tDCS	Anodal tDCS	Time		Group		Time by group	
	Adjusted mean (s.d.)^a^				*F*_(1,44)_	*p*^b^	*F*_(1,44)_	*p*^b^	*F*_(1,44)_	*p*^b^
Cognitive	*N* *=* 26	*N* *=* 24	*N* *=* 26	*N* *=* 24						
Simon Task										
Simon RT effect	45.78 (32.03)	59.91 (32.15)	49.92 (25.54)	58.86 (25.64)	0.11	0.74 (0.89)	2.52	0.12 (0.36)	0.32	0.58 (1.00)
Time Discrimination Task										
Total correct (%)	73.05 (12.31)	65.38 (16.17)	78.34 (11.78)	70.93 (15.47)	2.21	0.14 (0.84)	2.48	0.11 (0.45)	0.003	0.96 (1.00)
Macworth Clock Task										
Commissions (%)	4.50 (4.57)	4.91 (4.60)	5.09 (8.09)	4.80 (8.12)	2.42	0.13 (1.00)	0.001	0.93 (0.93)	0.25	0.62 (0.93)
Omissions (%)	34.26 (15.17)	38.82 (15.25)	27.24 (11.78)	34.14 (11.85)	1.79	0.19 (0.46)	2.89	0.10 (0.60)	0.26	0.61 (1.00)
Wisconsin Card Sorting Task (errors)										
Non-preservative	7.11 (4.89)	8.9 (4.89)	7.11 (3.57)	7.59 (3.72)	1.83	0.18 (0.54)	1.21	0.28 (0.48)	0.88	0.35 (1.00)
Preservative	12.20 (4.64)	13.53 (4.65)	13.39 (5.87)	13.32 (5.89)	0.503	0.48 (0.82)	0.27	0.61 (0.73)	0.51	0.48 (1.00)
List Sort Working Memory Task										
Total score	27.57 (14.20)	28.35 (14.65)	31.27 (16.65)	31.79 (17.18)	0.14	0.71 (0.95)	0.03	0.86 (0.94)	0.002	0.97 (0.97)
Verbal Fluency Task										
Letter % Corr	96.92 (3.97)	94.28 (3.98)	97.13 (4.08)	96.93 (4.09)	0.27	0.60 (0.90)	2.47	0.12 (0.29)	2.52	0.12 (0.72)
Semantic % Corr	96.24 (3.69)	97.08 (3.70)	98.08 (5.32)	94.89 (5.33)	0.12	0.74 (0.81)	1.22	0.28 (0.56)	5.89	**0.02** (0.24)
Speed of Processing										
MRT	382.86 (31.02)	393.21 (31.12)	361.39 (31.32)	367.46 (31.42)	1.97	0.17 (0.68)	1.16	0.29 (0.43)	0.17	0.68 (0.91)
Response Variability										
ICV	0.27 (0.00)	0.28 (0.00)	0.25 (0.00)	0.26 (0.00)	1.09	0.30 (0.60)	0.78	0.38 (0.51)	0.06	0.81 (0.97)
Prematurity										
Premature Resp.	13.55 (4.10)	24.99 (4.28)	7.78 (1.85)	8.39 (1.93)	0.10	0.75 (0.75)	3.32	0.08 (0.96)	2.46	0.12 (0.48)
Clinical										
Conners 3-P ADHD Index	8.01 (4.41)	12.53 (4.42)	11.72(4.94)	11.22 (4.95)	0.001	0.97 (0.97)	2.88	0.10 (0.30)	13.73	**0.001 (0**.004)
ARI										
Parent	0.72 (0.47)	0.79 (0.44)	0.67 (0.45)	0.49 (0.42)	0.27	0.61 (0.81)	2.77	0.10 (0.20)	2.89	0.20 (0.10)
Child	0.56 (0.29)	0.55 (0.5)	0.61 (0.40)	0.60 (0.4)	0.702	0.41 (0.82)	0.02	0.89 (0.89)	0.001	0.99 (0.99)
MEWS	16.16 (6.22)	16.04 (6.23)	18.08 (6.92)	14.87 (6.94)	5.01	**0.03** (0.12)	1.08	0.31 (0.37)	2.18	0.20 (0.15)
WREMB-R	15.21 (6.09)	18.28 (6.10)	n/t	n/t	n/a	n/a	2.96	0.09 (0.54)	n/a	n/a
CIS	16.75 (8.55)	19.82 (8.56)	n/t	n/t	n/a	n/a	1.52	0.22 (0.33)	n/a	n/a
Safety^c^										
Side effects	11.92 (7.02)	14.59 (7.03)	n/t	n/t	n/a	n/a	1.68	0.20	n/a	n/a
Adverse effects	15.04 (2.20)	16.33 (2.20)	n/t	n/t	n/a	n/a	**4.09**	**0.05**	n/a	n/a

ARI, Affective Reactivity Index; CIS, Columbia Impairment Scale-Parent; Corr, Correct; ICV, intrasubject coefficient of variance; MEWS, Mind Excessively Wandering Scale; MRT, mean reaction times; Resp, responses; s.d., standard deviation; WREMB-R, Weekly Parent Ratings of Evening and Morning Behaviour-Revised.

Benjamini–Hochberg adjusted *p* values given in parentheses.

a Adjusted values as predicted by the repeated-measures ANCOVA testing group differences at posttreatment and follow-up, adjusting for baseline, age at entry and medication status (naïve, off-medication, on-medication).

b Benjamini–Hochberg adjustment was applied to *p* values for time, group and time by group interaction effects separately and was applied separately to primary cognitive, secondary cognitive and secondary clinical outcome measures separately.

c Benjamini–Hochberg adjustment was not applied to these measures.

#### ADHD symptoms and related impairments

There was only a significant time by group interaction for Conners 3-P ADHD Index (*F*_1,44_ = 13.726, *p* = 0.004, *η*p^2^ = 0.238). Simple effects analysis showed significantly higher scores following anodal *v.* sham tDCS at posttreatment only [*p* = 0.001 (95% CI 1.92 to −7.11); follow-up: *p* = 0.73 (95% CI −3.39 to 2.41)] (Table 3).

### Exploratory analyses

Separate repeated-measures ANOVAs showed an effect of time (baseline *v.* posttreatment or follow-up) across groups. In cognitive measures, both groups improved in GNG, Simon, Letter Fluency tasks (baseline *v.* posttreatment or follow-up); Speed of Processing (baseline *v.* posttreatment only); and CPT % Omissions and Commissions, Mackworth Clock % Omissions, WM, Response Variability, and Prematurity (baseline *v.* follow-up only) (online Supplementary Table S9). In clinical measures, both groups improved in ADHD-RS and Conners 3-P Index (baseline *v.* posttreatment or follow-up); and in ARI Parent and Child, WREMB-R, and CIS (baseline *v.* posttreatment only).

Group differences in CT performance across the 3 weeks were explored with repeated-measures ANCOVAs covarying for baseline, age, medication status and total time spent playing each game. There were no significant effects after adjusting for multiple testing (online Supplementary Table S10).

We also explored if outcome changes that showed a significant time effect across both groups from baseline to posttreatment or follow-up were correlated with changes in CT performance scores (week 3 minus week 1). No correlations were significant (online Supplementary Table S11).

Given that age was not matched between groups, we conducted a post-hoc moderation analysis (Hayes, 2013), predicting a change in ADHD-RS (baseline minus posttreatment) in a regression analysis of stimulation group, age and a stimulation by age interaction. While the stimulation by age interaction was not significant [*β* = 0.2, s.e. = 0.12, *t*(46) = 1.7, *p* = 0.096], the simple effects showed a significantly reduced improvement for anodal tDCS *v.* sham in older participants [1s.d. above mean age; *β* = 9.53, s.e. = 4.26, *t*(46) = 2.24, *p* = 0.03], and an opposite but not significant pattern in younger children [1s.d. below mean age; *β* = −0.6, s.e. = 4.11, *t*(46) = −0.15, *p* = 0.88], indicating that older participants benefitted clinically less from anodal *v.* sham tDCS.

### Safety, feasibility, tolerability and blinding integrity.

There were no significant group differences in ratings of mood, wakefulness, overall impression of tDCS and CT (online Supplementary Tables S12 and S13), and side effects (Table 3). Adverse effects were significantly higher after anodal *v.* sham tDCS at posttreatment (*F*_1,44_ = 4.09, *p* *=* 0.05, *η*p^2^ = 0.08), driven mainly by higher parent-ratings for ‘he seems more grumpy and irritable’, ‘has little appetite’ and ‘has more problems falling asleep’ (Table 3). Stimulation was well tolerated, with only significantly higher reports of burning sensation during anodal than sham tDCS (online Supplementary Table S14). Group assignment guesses were not above chance for experimenters [χ^2^(1) = 3.9, *p* = 0.28], participants [χ^2^(1) = 1.85, *p* = 0.17] and borderline for parent guesses for anodal tDCS [χ^2^(1) = 3.57, *p* = 0.06], thus blinding was overall successful.

## Discussion

This double-blind, sham-controlled RCT administered 15 sessions of anodal or sham tDCS over rIFC combined with CT in 50 boys with ADHD, and found no improvement in ADHD symptoms or cognitive performance. Although both groups improved in clinical and cognitive measures over time, anodal relative to sham tDCS was associated with higher primary (ADHD-RS) and secondary (Conners 3-P ADHD Index) clinical outcome measures. Side effects did not differ, but at posttreatment, adverse effects relating to mood, sleep and appetite were higher following anodal than sham tDCS.

The lack of an observable clinical or cognitive effect extend previous meta-analytic evidence of no significant cognitive effects and limited evidence of clinical effects in ADHD with 1–5 anodal tDCS sessions over predominantly left dlPFC (Westwood et al., 2020). These findings are unexpected given that rIFC underactivation is consistently associated with poor cognitive control, attention and clinical symptoms in ADHD (Hart et al., 2013; Lukito et al., 2020; Norman et al., 2016; Rubia, 2018). While the findings of no clinical effect of tDCS of rIFC are novel, these extend evidence of no or moderate effects from prior 1–5 session sham-controlled tDCS studies stimulating rIFC in ADHD (see introduction) (Breitling et al., 2016, 2020; Soltaninejad et al., 2015a).

Findings are furthermore unexpected given evidence of a synergistic effect of combined CT and tDCS on improving cognition (Allenby et al., 2018; Au et al., 2016; Katz et al., 2017). Although we covaried for age, one possible explanation for the negative findings on clinical symptoms and cognition is that the anodal tDCS group were significantly younger with larger baseline clinical and cognitive impairments compared to sham, both of which could have impaired learning (Loe & Feldman, 2007). This is supported by evidence that ADHD children with worse neurocognitive skills at baseline show less CT gains (Minder, Zuberer, Brandeis, & Drechsler, 2019) and neurofeedback learning (Hammer et al., 2012; Lam et al., 2020; Zilverstand et al., 2017), while healthy controls with poorer cognitive performance or lower education achievement at baseline can nullify or lead to detrimental tDCS effects (Hsu, Juan, & Tseng, 2016; Jones & Berryhill, 2012).

Alternatively, given the stronger electric field strengths in children *v.* adults (Minhas, Bikson, Woods, Rosen, & Kessler, 2012; Salehinejad et al., 2020), multiple tDCS sessions may have triggered a homeostatic plasticity response – i.e. the amount and direction of plasticity was attenuated in response to excessive increases in neuronal excitability – thereby temporarily disrupting the excitability of rIFC (Fricke et al., 2011; Hoy & Fitzgerald, 2015; Karabanov et al., 2015; Wefelmeyer & Burrone, 2015). This is in line with our post-hoc moderation analysis that revealed older but not younger participants improved less in the ADHD-RS Total Scores in the anodal *v.* sham tDCS group at posttreatment. Future studies should verify if tDCS has differential effects depending on current strength and age of participants with ADHD.

Another possibility is that rIFC stimulation downregulated neighbouring dorsal prefrontal or parietal regions part of the dorsal attention network (Cubillo, Halari, Smith, Taylor, & Rubia, 2012; Hart et al., 2013), or left hemispheric prefrontal regions that mediate positive emotions (Gainotti, 2019; Groenewold, Opmeer, de Jonge, Aleman, & Costafreda, 2013).

Interestingly, however, impulsiveness/hyperactivity symptoms, which are most closely associated with rIFC activation (Rubia, 2018; Rubia, Smith, Brammer, Toone, & Taylor, 2005), were lower at follow-up after anodal relative to sham tDCS. This finding – that needs replication – could suggest longer-term neuroplastic consolidation effects as have been shown in other neurotherapies, such as neurofeedback (Alegria et al., 2017; Enriquez-Geppert, Smit, Pimenta, & Arns, 2019).

Both groups improved in symptoms and cognitive performance from baseline to posttreatment or follow-up, which could suggest gains due to CT (Wexler et al., 2016); however, given the lack of correlation with CT performance, placebo effects cannot be ruled out.

The negative findings from this trial are crucial given that tDCS is being increasingly incorporated into clinical practice, is considered an acceptable alternative to medication by parents, and is already commercially available (Buchanan, D'Angiulli, Samson, Maisonneuve, & Robaey, 2020; Sierawska et al., 2019). Particularly alarming is that parent-rated ADHD symptoms and adverse effects were higher at posttreatment after anodal tDCS relative to sham.

Findings are not encouraging for the efficacy of multi-session tDCS of rIFC combined with CT in ADHD. However, there are limitations. Although our sample of 50 participants is the largest sample of any tDCS study in paediatric ADHD, recent meta-analyses report relatively small cognitive effects of tDCS in ADHD (Westwood et al., 2020), suggesting that larger samples may be required to detect tDCS effects. Unfortunately, despite randomisation, groups were not balanced in several baseline measures which could have confounded the tDCS effects. However, measures that differed (i.e. clinical scores, performance, age and medication status) were covaried, minimising any confounding effects. Also, the anodal group had higher ODD symptoms at baseline, which could have affected emotional lability and confounded tDCS effects. However, no group differences were observed in changes of emotional dysregulation as measured on the ARI or in mood ratings. The findings in this study are limited to male adolescents with ADHD and the adopted protocol, and cannot be generalised to other populations (e.g. females; adults) or other protocols. Although we applied the largest number of sessions with the view of boosting consolidation of tDCS effects, the optimal number of sessions is not known in ADHD, leaving the possibility that fewer or even more sessions would optimise the tDCS effect (Au et al., 2016; Katz et al., 2017). Computational current flow models suggest higher stimulation intensities might be required to modulate clinical symptoms and cognitive functions mediated by rIFC given this is a deeper region compared to the commonly stimulated dlPFC (Salehinejad et al., 2020). Further, this study stimulated ‘F8’ in line with other studies (Breitling et al., 2016, 2020; Campanella et al., 2016, 2018; Dambacher et al., 2015; Soltaninejad et al., 2015a); however, improved performance on inhibitory control tasks in healthy adults has been reported when stimulating T4-Fz and F8-Cz intersection (Cunillera, Brignani, Cucurell, Fuentemilla, & Miniussi, 2016; Jacobson, Javitt, & Lavidor, 2011, 2012; Stramaccia et al., 2015) or F6 (Cai et al., 2016; Gómez-Ariza, Martín, & Morales, 2017; Hogeveen et al., 2016; Sallard, Mouthon, De Pretto, & Spierer, 2018), which cover the rIFC along with areas closer to the surface implicated in motor inhibition (e.g. superior and middle frontal cortex, and the supplementary motor area) (Hart et al., 2013; McCarthy et al., 2014; Zhang, Geng, & Lee, 2017) and attention (e.g. right dlPFC, part of the dorsal attention network and typically underactivated in ADHD) (Cubillo et al., 2012; Hart et al., 2013). Another limitation is that we could not test for weekly dose effects as ADHD symptoms were only measured at baseline, posttreatment and follow-up; yet weekly changes in CT performance did not show dose effects.

Larger, double-blind, randomised-controlled trials should systematically investigate optimal and ideally individualised stimulation protocols (e.g. different stimulation sites, intensity, duration, number of sessions, etc.) measuring clinical, cognitive and possible non-targeted cognitive outcomes. Stimulating T4-Fz and F8-Cz intersection and F6 could potentially be more effective for improving inhibitory control and attention functions in ADHD.

## Conclusion

This rigorously conducted double-blind, randomised, sham-controlled trial of 15-weekday sessions of anodal tDCS over rIFC combined with CT in 50 boys with ADHD showed no clinical or cognitive improvement. Findings suggest that rIFC stimulation may not be indicated as a neurotherapy for cognitive or clinical remediation for ADHD.

## Footnotes

† The notes appear after the main text.

## Notes

1 The saline solution was held in *tufts* by capillary forces between strands, which prevented it from spreading/dripping.

## Supplementary material

For supplementary material accompanying this paper visit https://doi.org/10.1017/S0033291721001859. click here to view supplementary material
